# *In Vivo* hip joint loading during cross-country skiing on a simulator

**DOI:** 10.3389/fbioe.2025.1653208

**Published:** 2025-10-23

**Authors:** Daniela Aschenbrenner, Pamela Scupin, Jörn Dymke, Sijia Zhou, Tazio Maleitzke, Stefan Hainzl, Georg N. Duda, Carsten Perka, Bernd Wolfarth, Tobias Winkler, Philipp Damm

**Affiliations:** ^1^ Berlin Institute of Health at Charité – Universitätsmedizin Berlin, Julius Wolff Institute, Berlin, Germany; ^2^ Charité – Universitätsmedizin Berlin, Corporate Member of Freie Universität Berlin and Humboldt-Universität zu Berlin, Center for Musculoskeletal Surgery, Berlin, Germany; ^3^ Department of Orthopaedic Surgery, Trauma Orthopaedic Research Copenhagen Hvidovre (TORCH), Copenhagen University Hospital - Amager and Hvidovre, Hvidovre, Denmark; ^4^ Department of Clinical Medicine, University of Copenhagen, Copenhagen, Denmark; ^5^ Department of Sports Medicine, Charité – Universitätsmedizin Berlin, corporate member of Freie Universität Berlin and Humboldt-Universität zu Berlin, Center for Musculoskeletal Surgery, Berlin, Germany; ^6^ Department of Sports Medicine, Institute of Sports Science, Humboldt-Universität zu Berlin, Berlin, Germany; ^7^ Berlin Institute of Health Center for Regenerative Therapies, Berlin Institute of Health at Charité – Universitätsmedizin Berlin, Berlin, Germany

**Keywords:** cross-country skiing, diagonal poling, double poling, joint loading, downhill skiing, instrumented hip implants, *in vivo* hip joint loading, ski

## Abstract

**Background:**

Return to sports following total hip arthroplasty (THA) is important to an increasingly younger and active clientele. However, knowledge about *in vivo* joint forces during sports is scarce and often only estimated. As skiing is controversially debated as an activity following THA, we measured *in vivo* joint loads in instrumented THAs during cross-country ski simulation.

**Methods:**

Five untrained subjects who had previously received instrumented THAs were instructed to perform double poling and diagonal poling exercises on a ski simulator and to walk on a treadmill at 4 km/h as a reference exercise. The joint contact force, bending moment, and torsion torque on the implant were determined. Time-load patterns were generated. Loading peaks were compared intra-and inter-individually to walking. Statistical parameter mapping was used to visualise significant differences between exercises across the movement cycle.

**Results:**

Loading maxima were mostly lower or adjacent to loading maxima of walking, except for diagonal poling with foot lift. Differences in execution of double poling resulted in different time-load patterns of torsion torque. Diagonal bending moments exceeded walking bending moments slightly. Outliers were observed.

**Conclusion:**

Double or diagonal poling can be safely practiced by THA patients on a ski simulator in the late postoperative period due to mostly lower or adjacent loading forces to walking. Unilateral standing phases should be minimized. Patient’s experience and bone quality affect recommendation of this sport. Limitations concern limited generalizability of small cohort and simulated environment.

## 1 Introduction

Around 2.5 million individuals in the U.S. underwent total hip arthroplasty (THA) in 2010, with an increase of around one million per year (Maradit et al., 2015). The number of procedures is expected to rise further (Robinson et al., 2023; Saueressig et al., 2021) with increasingly younger recipients (Ollivier et al., 2012; Trousdale et al., 1999; Pabinger and Geissler, 2014). THA are expected to increase by 27% in 2040 (Pilz et al., 2018), which is attributed to an ageing society and rising obesity rates (Zhang et al., 2008). These factors increase the risk of developing osteoarthritis (OA), the most common indication for arthroplasty treatments (Robertsson et al., 2010). With an increasing demand for THA, more patients rightfully focus on an improved quality of life post-operatively. Thus, longevity and safety in THA must be optimized, while minimizing revision rates and implant failures to accommodate changing demographics of THA patients. Although surgical techniques and material innovations have improved outcomes following THA (Pilz et al., 2018), implant failures due to aseptic loosening, periprosthetic fractures, and mechanical wear have also increased (Oltean-Dan et al., 2022; Feyzi et al., 2021; Broomfield et al., 2017).

It was previously established that low-impact activities such as walking, cycling, and swimming, as well as postoperative early mobilization like full weightbearing physiotherapy do not increase the risk for implant failure (Fortier et al., 2021; Zhou et al., 2023; Damm et al., 2013; Damm et al., 2017) and aid muscle and tendon healing and bony ingrowth of joint implants likewise (Torcasio et al., 2008; Zhang et al., 2021). However, non-compliance with mobility restrictions especially in the first 6 months following THA increase the risk of implant dislocation (Karachalios et al., 2018). High impact sports induce mechanical wear (Ollivier et al., 2012).

Concerns about the safety of alpine or downhill skiing led to restricted recommendations from surgeons (McGrory et al., 1995; Swanson et al., 2009). A 2000 study by Gschwend et al. suggests more mechanical wear and mild periprosthetic osteolysis in very active skiers when compared to non-active skiers after THA(22). However, active skiing did not lead to higher risk of aseptic loosening (Gschwend et al., 2000) or revision rates (Lancaster et al., 2022). No *in vivo* data are currently available. The main method for loading assessment is musculoskeletal modelling. However, modelling consistently overestimates loading compared to *in vivo* data (Tomasi et al., 2023). Bogert et al. used accelerometers and muscle modelling to analyse hip joint loading during cross-country skiing. It led to higher loading values of ca. 4–5 times body weight compared to walking (Van den bogert et al., 1999). There are no further studies to our knowledge reporting hip joint loading during simulated or real world cross-country skiing. Our current *in vivo* loading data on cross-country ski simulation may help to outline evidence-based suggestions for patients regarding their preferred leisure activities.

## 2 Materials and methods

### 2.1 Participating subjects

Ten subjects were previously treated with instrumented THA (Damm et al., 2010; Bergmann et al., 2007; Graichen et al., 2007) due to severe OA. Six patients consented to participate in the skiing trials 10 years after implantation, of which five were able to execute at least two of the cross-country skiing movements correctly (Table 1). None of the participants reported any real world or simulated skiing experience. Subjects had no grave comorbidities and movement was ensured to be painless. Except for one patient (H8L), the instrumented joint was on the right side. For comparability, data from H8L were mirrored. The exercises were executed all in 1 day. Measurements took place in the years 2019–2022.

**TABLE 1:** Demographic characteristics of subjects.

Patients	Sex	Age	Bodyweight	Height	BMI	Time post-operation
		[years]	[kg]	[cm]	[kg/m^2^]	[months]
H2R	Male	73	88	172	30	146
H6R	Male	78	84	176	27	120
H7R	Male	62	93	179	29	120
H8L	Male	64	88	178	28	114
H10R	Female	62	94	162	36	110
Median	—	64	88	176	29	120

### 2.2 Ski simulator

The simulator ‘Thorax Trainer’ (Move Sports, Köln, Germany) was used to imitate low-impact cross-country skiing via a rail system and attached poles. The simulator does not imitate real world skiing and instead acts as a training tool for the coordination and muscles required in cross-country skiing. It allows training of the upper and lower body simultaneously, which is necessary for the choreography of cross-country skiing. Double-pole (DBP) and diagonal arm movement (DIAP) with corresponding lower limb movement can be trained and recorded. The resistance of the simulator was consistently set as low as possible.

### 2.3 Exercises on the simulator

**FIGURE 1:**
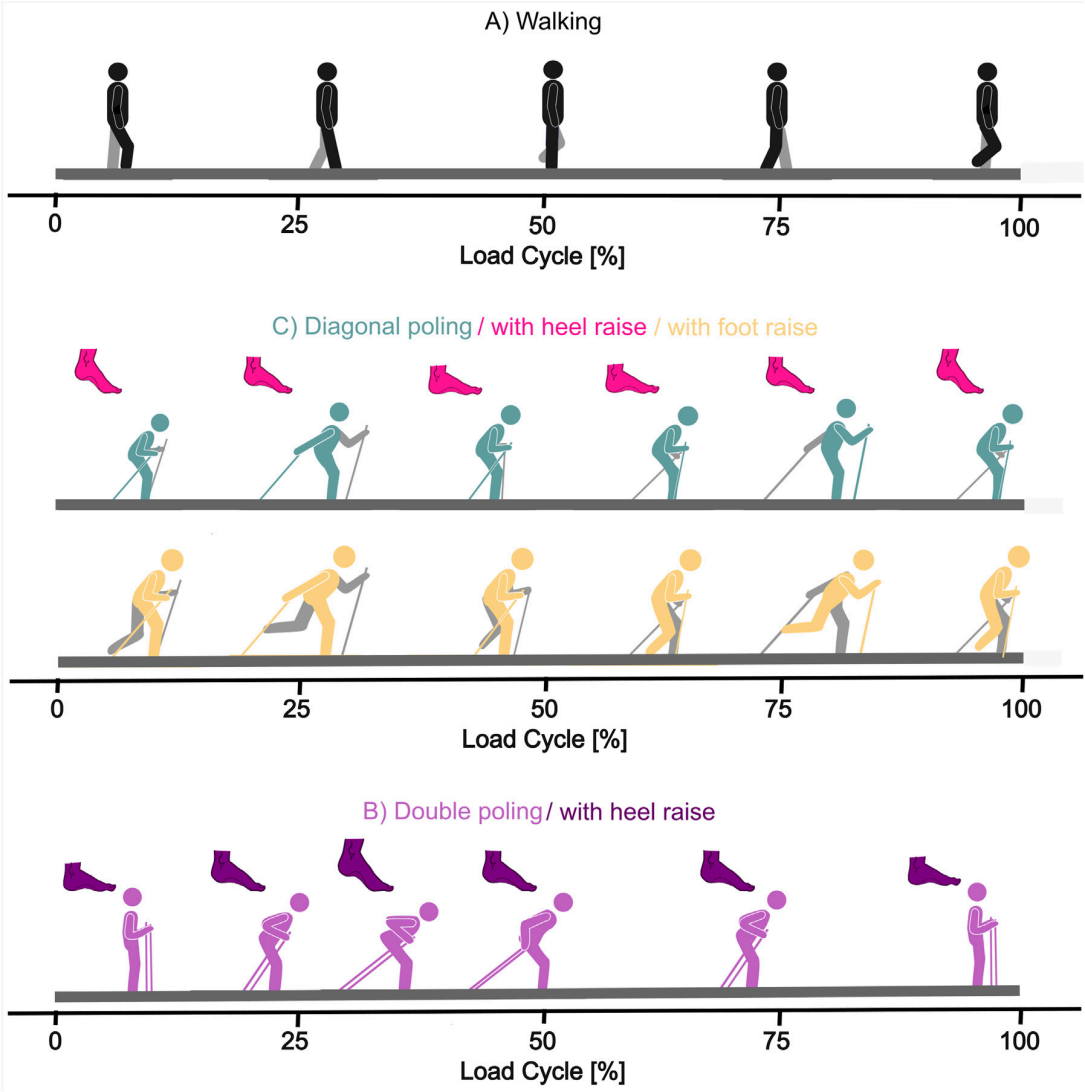
Exercises on the simulator as movements according to the load cycle. Colored parts symbolize the instrumented side, grey parts the contralateral side. Mannequins in **(A)** demonstrate walking: The second pictogram is at ipsilateral heel strike (25% load cycle) and the third is at ipsilateral heel off (75% load cycle). **(B)** Shows double poling without (light purple mannequin) and with heel raise (dark purple foot + light purple mannequin). **(C)** demonstrates diagonal poling without (green mannequin), with heel raise (pink foot + green mannequin), or leg raise (yellow mannequin). Pole use is equal in all variations with only leg or foot movements differing. The second mannequin in **(C)** shows maximal ipsilateral thrust, and the fifth shows maximal contralateral thrust.

All participants practiced for 5 minutes before each exercise to get acquainted with the simulators and movements. Each activity was repeated at least eight times. Walking on a treadmill at 4 km/h was used as the reference activity (Figure 1A). The movement cycle starts with an ipsilateral leg swing phase until ipsilateral heel strike. Shortly after heel strike, the ipsilateral standing phase begins and ends after ipsilateral toe-off. The datasets for skiing techniques used and analysed during the current study are available from the corresponding author on reasonable request. Datasets for walking on a treadmill at 4 km/h are available in the Orthoload database^1^.

Using the cross-country ski simulator, double poling (DBP) and diagonal pole use (DIAP) could be tested. Participants stood on a treadmill with mobile poles attached to the sides which could be moved back and forth.

Two double poling exercises were constituted (Figure 1B): In the first exercise, a movement cycle was started by a backwards pole thrust initiating the verve phase with simultaneous flexion of both hips and knees, followed by a swing phase that led the poles to the front again and upright body position (DBP). In the second exercise, a heel lift in the verve phase was added (DBPwH). Three exercises involved diagonal poling (Figure 1C): In all of them, a movement cycle began with the ipsilateral pole use and thrust, followed by contralateral pole use and thrust. Ipsilateral thrust happened simultaneously to contralateral forward swing and vice versa. In DIAP, participants performed the exercise solely with flexion of hips and knees and without any lifting of the feet. In the second exercise, the heel on the same side of the thrusting arm had to be lifted (DIAPwH). In the third exercise, the whole foot on the side of the thrusting arm had to be lifted, which was achieved by a flexion of the knee and slight extension of the hip (DIAPwF).

### 2.4 Instrumented hip implants and *in vivo* measurement

**FIGURE 2:**
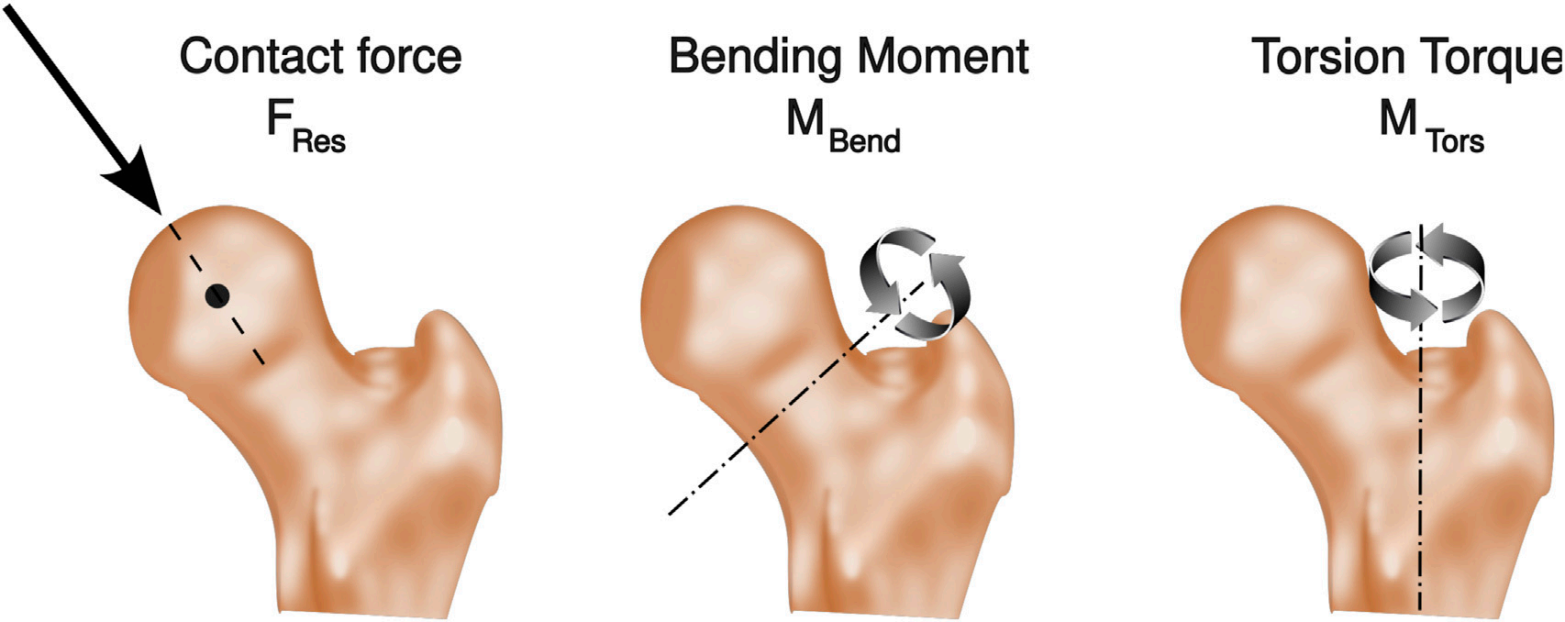
*In vivo* joint loads. Resultant joint contact force (FRes) in the hip joint, the bending moment at the femur neck (M_Bend_), and the torsion torque around the femur stem (M_Tors_).

The subjects received an uncemented hip implant capable of telemetric data transmission, as has been described previously (Damm et al., 2010; Heinlein et al., 2007). The resultant joint contact force (F_Res_) was measured directly by six strain gauges in the hollow neck of the implant (Wu et al., 2002) and transmitted to an external device (TELEPORT, Orthoload, 2025). Bending moment (M_Bend_) acting on the femoral neck, and torsion torque (M_Tors_) acting around the femur shaft were calculated based on the measured joint contact forces, the implant geometry and their orientation in the femur (Damm and Bender, 2023) (Figure 2). All values were normalized to the patients’ body weight with units of % BW and % BWm for moments respectively.

### 2.5 Analysis of time-load patterns

Because the timing and magnitudes of loading maxima are prognostic for implant endurance, subject-specific time-force respectively time-momentum curves were generated. But since ‘typical’ time-load patterns cannot simply be achieved by calculating arithmetic mean, a dynamic time-warping (DTW) algorithm was used to account for timing differences between the repetitions of an exercise (Bender et al., 2012; Bender and Bergmann., 2025). The ability to correctly execute the exercises varied extensively between subjects and only correct movements were included. Hence the number of cycles included in this calculation varied between eight and 56 cycles for an exercise *per* subject. To generate median time-load curves, time-dependent loads and moments have again been subjected to the dynamic time-warping algorithm and the resulting time-load curves were analyzed descriptively for each exercise.

### 2.6 Statistical analysis

All *in vivo* measured forces and moments are specified in relation to body weight (% BW) respectively % BW*meter (% BWm) to account for inter-individual weight differences. Intra-individual analysis involved Statistical Parametric Mapping (SPM) to compare every data point of one exercise to the according data points of a comparable exercise via dependent t-test. This way, significant differences throughout the whole movement cycle could be found. Additionally, peak values of each exercise were compared intra- and inter-individually to peak values during walking using the t-test for dependent variables. We focused on descriptive statistics by reporting ranges of maxima and interquartile ranges due to the few participants. R version 4.3.1 was used for statistical analysis of median maxima and generation of time-load curves. The package spm1d version M.0.4.10 for MATLAB was used for intra-individual SPM-Analysis. p < 0.05 was considered significant.

## 3 Results

### 3.1 *In vivo* time-load curves

#### 3.1.1 Walking

**FIGURE 3:**
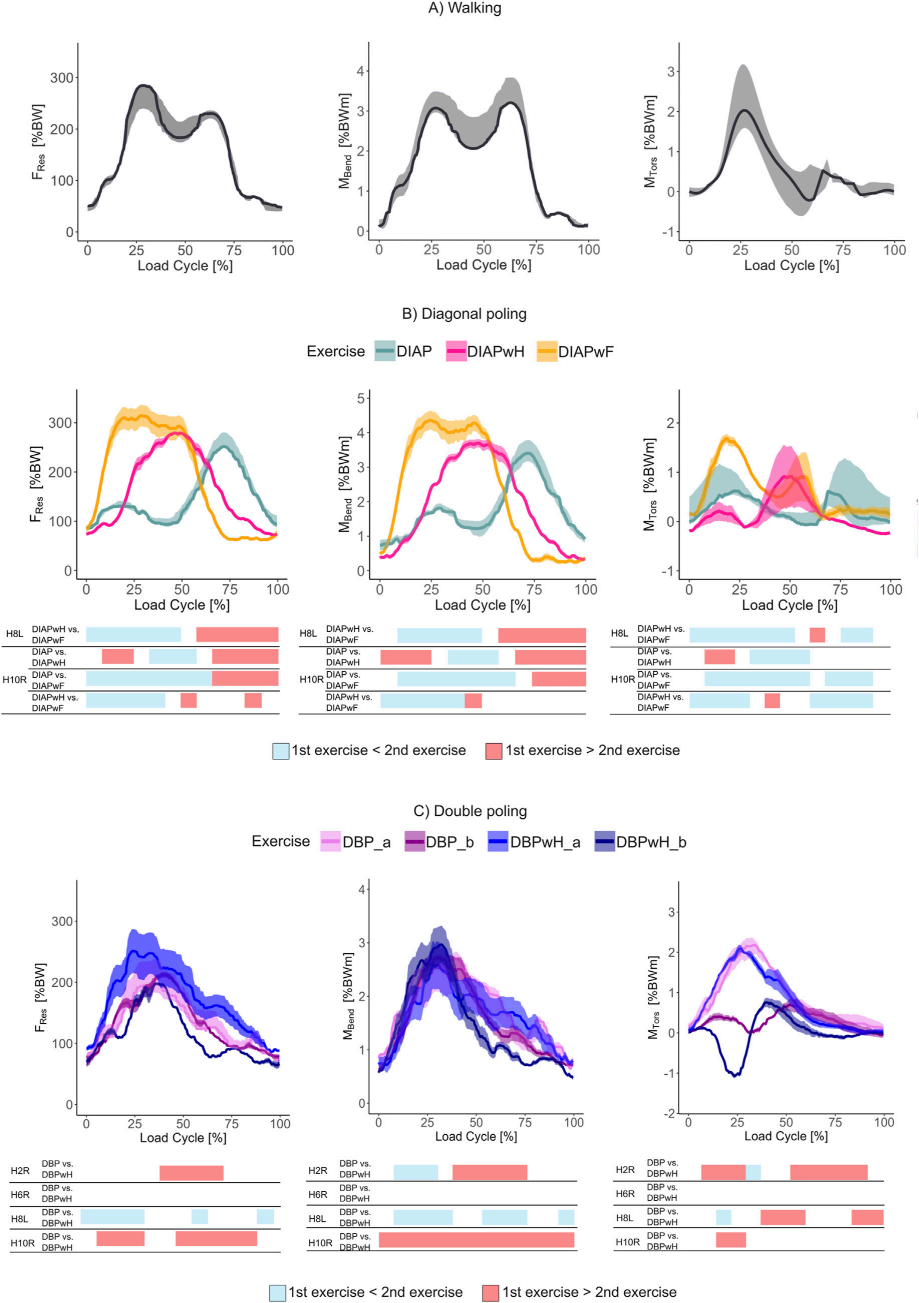
*In vivo* hip joint loading profile during **(A)** Walking, **(B)** Diagonal poling, and **(C)** Double poling. Average time-load patterns of F_Res_, M_Tors_, M_Bend,_ and their 25th and 75th percentiles (shaded areas). F_Res_ = resultant joint contact force, M_Tors_ = torsion torque around the femoral shaft axis, M_Bend_ = bending moment at the middle of the femoral neck. Forces in percent of body weight (% BW) and moments in percent of body weight × meter (% BWm). SPM results as color-coded tables are below each graph: Subjects are listed in the first column, compared exercises in the second. Light blue areas signify periods, in which the first-mentioned exercise of the second column is significantly lower than the second exercise; vice versa for red areas.

All subjects were able to complete 20 cycles of walking on a treadmill at 4 km/h. The average time-load curve of all subjects resulted in similar two-peaked curves, with peaks at heel strike (25%–30% load cycle) and toe-off (65%–70% load cycle, Figure 3A). F_Res_ and M_Tors_ were highest at heel strike (median F_ResMax_ = 284% BW, median M_TorsMax_ = 2.04% BWm). The bending moment was highest at toe off (median M_BendMax_ = 3.06% BWm).

#### 3.1.2 Diagonal poling

Diagonal poling (DIAP) was performed by three subjects correctly. Two subjects were not able to coordinate the pole and leg movement according to protocol and were excluded from analysis. The time-load curves showed two peaks at times of either maximal pole thrust (20%–30% load cycle = maximal ipsilateral pole thrust, 70%–80% load cycle = maximal contralateral pole trust, Figure 3B). The second peak shows the highest results for all parameters (median F_ResMax_ = 251% BW, median M_BendMax_ = 3.41% BWm, median M_TorsMax_ = 0.62% BWm).

Diagonal poling with heal lift (DIAPwH) was correctly performed by H8L and H10R. Load maxima happened at around 50% load cycle for all parameters during the ipsilateral standing phase (median F_ResMax_ = 279% BW, M_BendMax_ = 3.70% BWm, median M_TorsMax_ = 1.40% BWm).

Diagonal poling with foot lift (DIAPwF) was again correctly performed only by H8L and H10R. It resulted in a loading plateau for the first half of movement for F_Res_ and M_Bend_ during ipsilateral standing (median F_ResMax_ = 315% BW, median M_BendMax_ = 4.61% BWm). During the start and end of the ipsilateral standing phase, two respective peaks in torsional torque appeared (median M_TorsMax_ = 1.90 % BWm).

**FIGURE 4:**
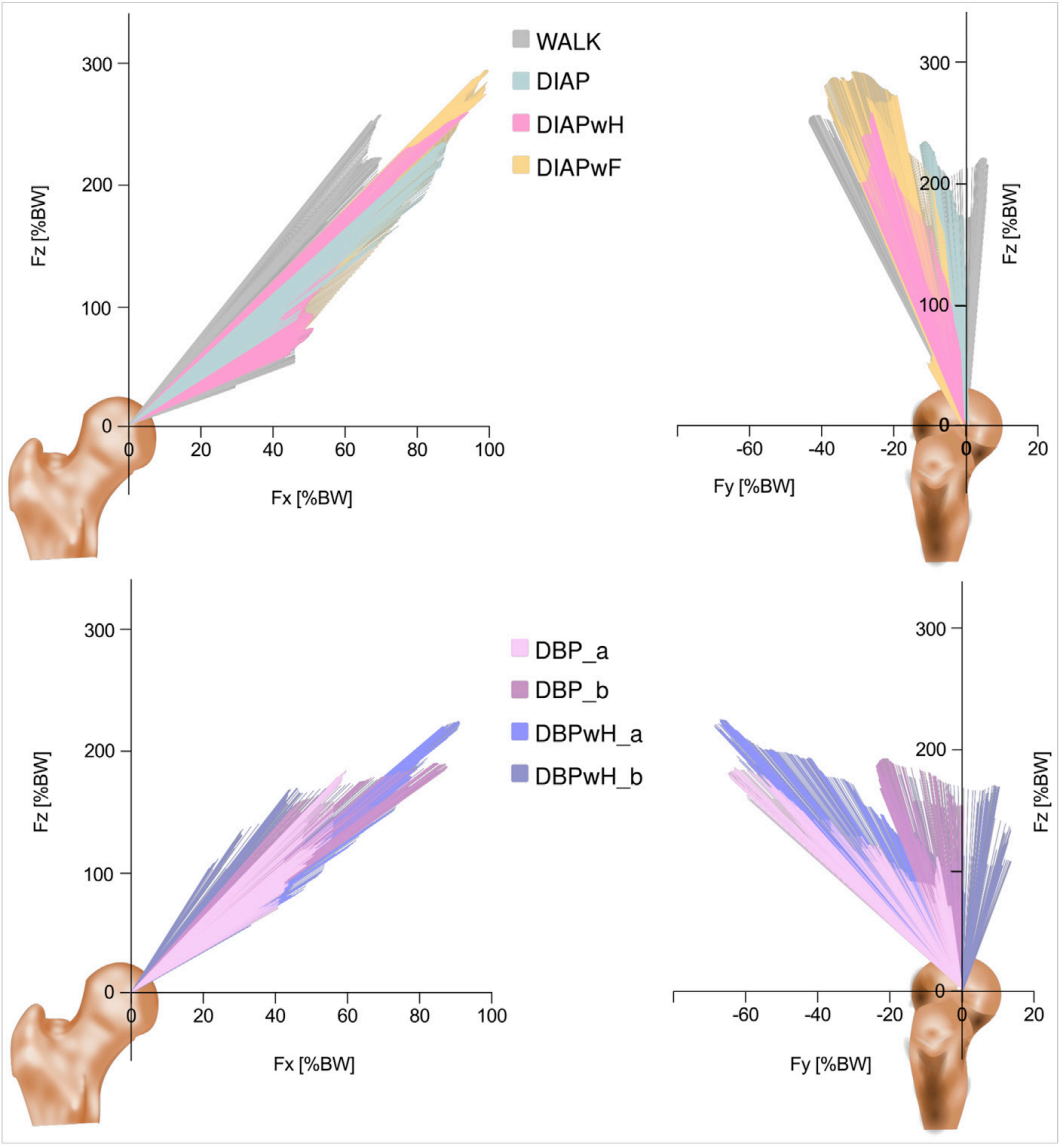
Vector plots of resultant contact force for double poling (top) and diagonal poling (bottom) exercises in accordance with the femur head. Left: Frontal plane. Right: Sagittal plane. Fz = Vertical axis, Fx = Frontal axis, Fy = Saggital axis. Forces in percent of body weight (% BW). DBP = Double poling, DBPwH = Double poling with heel lift, DIAP = Diagonal poling, DIAPwH = Diagonal poling with heel lift, DIAPwF = Diagonal poling with foot lift, WALK = walking on a treadmill (4 km/h).

The resultant force vectors for each exercise are shown in Figure 4.

#### 3.1.3 SPM-analysis of diagonal poling

Intra-individual comparison of H8L and H10R confirmed that DIAPwF reached significantly higher loading for all parameters in the first 50% of movement during ipsilateral standing compared to DIAPwH and vice versa. Only H10R could perform all diagonal poling exercises. During DIAP, loading was significantly lower than both other exercises. However, during either maximal pole thrust, the loading exceeded that of DIAPwH. During the second peak at ipsilateral weight shift, it exceeded both DIAPwH and DIAPwF significantly.

#### 3.1.4 Double poling

DBP was performed by all subjects correctly. H7R was not able to coordinate the additional heel lifts during DBPwH, so we excluded them from analysis for DBPwH. DBP and DBPwH produced similar time-load curves for all parameters: F_Res_ and M_Bend_ rose to the point of the deepest squatting position at 20%–30% load cycle (Figure 3C, DBP: median F_ResMax_ = 203% BW, median M_BendMax_ = 2.71% BWm. DBPwH: median F_ResMax_ = 199% BW, median M_BendMax_ = 2.88% BWm). Stratification was apparent in M_Tors_: Group “a”, consisting of H6R, H7R (not for DBPwH) and H8L showed a rise in M_Tors_ at the time of squatting, while group “b”, consisting of H2R and H10R exhibited a negative or low value during full squat (DBP: median M_TorsMax_(a) = 2.18% BWm, median, M_TorsMax_(b) = 0.76% BWm. DBPwH: median M_TorsMax_(a) = 2.22% BWm, median M_TorsMax_(b) = −1.10% BWm).

#### 3.1.5 SPM-analysis of double poling

Intra-individual SPM comparison of DBP and DBPwH showed higher loading for DBP on multiple occasions for subjects H2R and H10R. H8L on the contrary had lower loading during DBP than DBPwH except for M_Tors_. Both exercises did not differ significantly in the case of H6R in terms of loading. SPM-Analysis could not be applied to H7R, because they did not perform DBPwH.

#### 3.1.6 Intra-individual comparison between exercises and walking

The load maxima of every exercise were compared to the load maximum during walking for each subject individually. DBP and DBPwH resulted in lower peak loading than walking. This applies to every subject and parameter significantly, including some exceptions (Table 2). DBP led to a median lowering of hip joint loading of 33% F_ResMax_, 30% M_BendMax,_ and 13.25% M_TorsMax_ compared to walking (only significant differences were used to calculate the median difference). Only H8L had higher values during DBPwH than walking: F_ResMax_ was 13% and M_TorsMax_ 67% higher (*p* < 0.001). No significant difference was found in M_BendMax_.

**TABLE 2:** Intra-individual comparison of median maximum values of resultant force (F_Res_), bending moment (M_Bend_) and torsion torque (M_Tors_) for cross-country skiing and walking.

Subject	Parameter [% BW]	Walk vs. DBP		Walk vs. DBPwH		Walk vs. DIAP		Walk vs. DIAPwH		Walk vs. DIAPwF	
		Δ [%]	p	Δ [%]	p	Δ [%]	p	Δ [%]	p	Δ [%]	p
H2R	FResMax	−15	<0.001	−18	<0.001	32	<0.001				
H6R	FResMax	−37	<0.001	−35	<0.001						
H7R	FResMax	−33	<0.001			−15	<0.001				
H8L	FResMax	−2	0.228	13	<0.001			−7	0.039	27	<0.001
H10R	FResMax	3	0.369	−6	0.030	13	<0.001	32	<0.001	28	<0.001
Median	FResMax	−33		−12		13		13		28	

Subject	Parameter [% BWm]	Δ [%]	p	Δ [%]	p	Δ [%]	p	Δ [%]	p	Δ [%]	p
H2R	MBendMax	7	0.174	24	<0.001	39	<0.001				
H6R	MBendMax	−49	0.001	−45	<0.001						
H7R	MBendMax	−44	<0.001			−1	<0.001				
H8L	MBendMax	−14	<0.001	1	0.827			−2	0.578	−94	<0.001
H10R	MBendMax	−16	<0.001	−17	<0.001	6	0.033	45	<0.001	43	<0.001
Median	MBendMax	−30		−17		6		45		−25	

Subject	Parameter [% BWm]	Δ [%]	p	Δ [%]	p	Δ [%]	p	Δ [%]	p	Δ [%]	p
H2R	MTorsMax	54	<0.001	−48	<0.001	−41	<0.001				
H6R	MTorsMax	−38	<0.001	−31	0.002						
H7R	MTorsMax	−13	<0.001			−20	<0.001				
H8L	MTorsMax	55	<0.001	67	<0.001			−53	<0.001	29	<0.001
H10R	MTorsMax	−28	0.040	−9	0.099	12	0.043	236	<0.001	204	<0.001
Median	MTorsMax	−13		−19		−20		92		116	

Δ = difference in %. Only significant values were used for median. Independent t-test, p < 0.05 deemed significant. Activities: Walk = Walking on treadmill (4 km/h), DBP = Double poling, DBPwH = Double poling with heel lift, DIAP = Diagonal poling, DIAPwH = Diagonal poling with heel lift, DIAPwF = Diagonal poling with foot lift, DIAPwF = Diagonal poling with foot lift.

DIAP had slightly higher hip joint loading of 13% F_ResMax_ and 6% M_BendMax_ compared to walking, but 20% lower M_TorsMax_. During DIAPwH, the two subjects showed divergent trends: H8L showed lower values for F_ResMax,_ M_BendMax_ and M_TorsMax_ (−7%, −2%, −53%) compared to walking, while H10L showed much higher loading, especially for M_Tors_ (236%). DIAPwF led to higher values compared to walking for all loading parameters for both these subjects except M_BendMax_ of H8L. H10R had a 204% greater M_TorsMax_ than walking and is by far the greatest difference compared to any other parameter, patient and exercise.

### 3.2 Inter-individual comparison between exercises and walking

**FIGURE 5:**
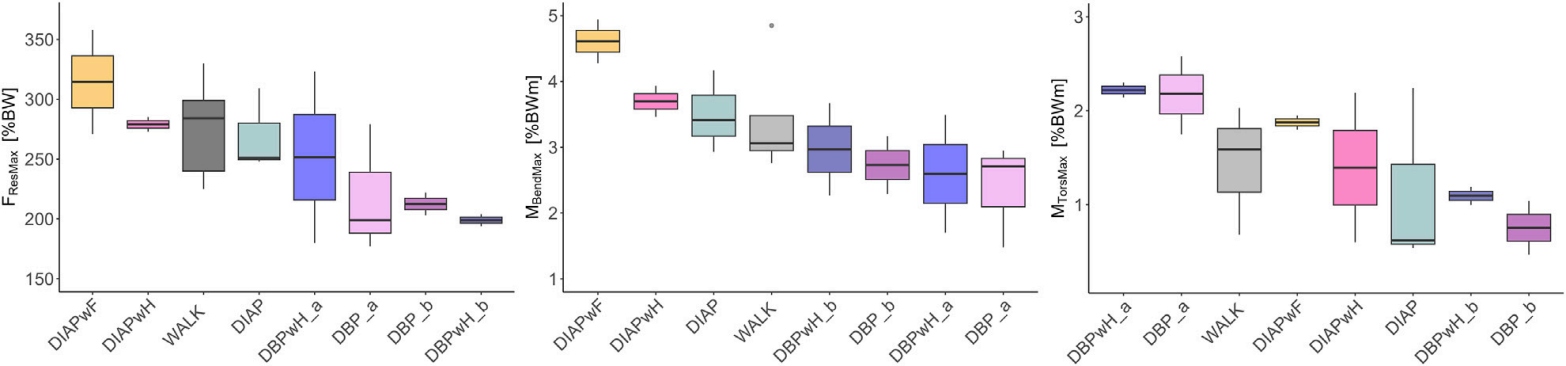
Inter-individual comparison of maximum loading values, interquartile ranges (coloured boxes, quartile 25%, 75%) of walking and cross-country skiing exercises. FResMax, the absolute maximum of resultant *in vivo* contact force; M_TorsMax_, the absolute maximum of *in vivo* torsional moment around the femoral shaft axis; M_BendMax_, the absolute maximum of resultant *in vivo* bending moment at the middle of the femur neck. Forces in percent of body weight (% BW) and moments in percent of body weight × meter (% BWm). Activities: DIAPwF = Diagonal poling with foot lift; DBP = Double poling; DBPwH = Double poling with heel lift; DIAP = Diagonal poling; DIAPwH = Diagonal poling with heel lift; DIAPwF = Diagonal poling with foot lift.

We compared the median maxima of every exercise and walking and report their range. DIAPwF exceeded the F_ResMax_ of walking slightly (Figure 5). They ranged from 271 to 358% BW and 225–330% BW respectively. Both double poling exercises had the lowest F_ResMax_. They ranged from 177 to 279% BW for DBP and 180–323% BW for DBPwH. All diagonal poling varieties showed higher M_BendMax_ than walking, led by DIAPwF and followed by DIAPwH and DIAP [range: 4.28–4.94% BWm, 3.46–3.93% BWm, 2.93–4.17% BWm]. The walking range was 2.76–4.85% BWm. Lastly, the M_TorsMax_ of the subgroup “a” of DBP and DBPwH exceeded walking and all other exercises [range: 1.75–2.58], in contrast to “b”, which had the lowest M_TorsMax_ of all exercises [range: 0.47–2.3]. The walking range was 0.68–3.22% BWm.

## 4 Discussion

To our knowledge, we present the first *in vivo* study on hip joint loading during cross-country skiing performed on a simulator. We found out that DBP had a lower peak contact force of 203% BW and a bending moment of 2.71% BWm (both groups included) compared to walking. Through SPM analysis it was apparent that an extra heel lift did not lead to higher loading throughout the cycle. We found two main groups when we analyzed time-load patterns of torsion torque: While one group showed higher torques during both double poling varieties, the second showed negative or very low torques. We found the first group to have a much less pronounced squat, and a more forward-leaning upper body posture, hence greater force vector orientation towards the sagittal axis. The second group’s execution might be considered backward-leaning with a deep squat technique. Both techniques are valid, but they serve slightly different functions: A greater squat with upright body position is seen in more experienced skiers and during uphill skiing (Zhang et al., 2008; Choi et al., 2021; Stöggl and Holmberg, 2016), but less knee flexion can cause less exhaustion and more durability (Damm et al., 2017; Holmberg et al., 2006). It was the first group with less knee flexion which exhibited higher torsion torques compared to the second.

Diagonal poling varieties resulted in similar or slightly higher loading maxima compared to walking, torsion torque excepted. This was especially the case for bending moment, where any variety exceeded walking maxima. Weight shifts to one leg or unilateral standing pose the greatest risk for increased loading. Repeatedly high bending moments can lead to implant failures like loosening or periprosthetic fractures (Lakstein et al., 2011), so it is important to include personal risk factors like osteoporosis before recommending diagonal poling on the ThoraxTrainer (Sidler-Maier and Waddell, 2015).

Despite relatively low peak contact force during DIAP, SPM analysis showed significantly higher bending and contact force at the time of the ipsilateral pole thrust. During this phase, weight is shifted toward the according leg. Through SPM, it is possible to evaluate prolonged phases of increased loading, even if the maximum load is not high comparatively. However, in this trial, SPM results showed a relatively broad variety between subjects: For example, H6R had no significant loading differences between DBP and DBPwH. H2R and H10R had significantly higher loading during DBP than DBPwH. For H8L, it was the opposite. These findings suggest that subjects executed the same exercises differently, which may be attributed to their previous lack of experience with any skiing technique. Given the low number of participants, it limits the generalizability of this study. Accordingly, physicians should take a THA patient’s previous experience with skiing techniques into account before recommending cross-country ski simulation.

Among the exercises that have been analyzed in studies for *in vivo* hip joint loading, jogging at 5 km/h exceeded all cross-country ski simulator exercises (Bergmann et al., 1993) with F_ResMax_ = 480% BW, M_BendMax_ = 7.55% BWm and M_TorsMax_ = 3.95% BW. Some daily activities exceed the loadings in this study, as is the case for downstairs walking (Bergmann et al., 1995; Kutzner et al., 2010) with F_ResMax_ = 387% BW, M_BendMax_ = 6.65% BWm and M_TorsMax_ = 2.7% BWm. Even one physiotherapeutic exercise in early postoperative rehabilitation can have higher loads: One-legged bridging (Schwachmeyer et al., 2013) led to F_ResMax_ = 303% BW, M_BendMax_ = 2.2% BWm and M_TorsMax_ = 4% BWm. While jogging is not commonly recommended, downstairs walking and one-legged bridging are usually not discouraged. However, some other sports like swimming and ergometer cycling have lower loads with F_ResMax_ = 193% BW, M_BendMax_ = 1.27% BWm, and M_TorsMax_ = 0.82% BWm in the case of breaststroke swimming (Zhou et al., 2023; Damm et al., 2017).

One limitation of our study is the limited generalizability. The patient cohort was mostly male (4:1 male-to-female ratio) and all participants were over 60 years old. As the only female participant and the only participant with an obese BMI, H10R exhibited outlier values for DIAPwF and DIAPwH. The intra-individual difference in M_Tors_ was over 200% compared to walking. This might be a sex-dependent effect of different hip joint anatomy and kinematics (Boyer et al., 2008; Czuppon et al., 2017) and/or related to their BMI(45) All subjects were previously untrained in skiing techniques. They received training for the techniques immediately before data collection. In contrast to H7R, the two subjects H8L and H10R had little difficulties learning most techniques. The exhaustion levels likely varied between participants. Exhaustion was not quantified and its effect could not be evaluated in terms of loading. It can be assumed from previous investigations that a better muscle status, correlating with a younger age, would lead to even lower hip joint loading than what has been found in this study (Damm et al., 2019; Mikesky et al., 2000).

Caution is warranted when extrapolating these findings to on-snow performance: Pannizzollo et al. have shown that real skiing resulted in higher EMG activities of M. quadriceps femoris than simulated skiing (Panizzolo et al., 2013). In real skiing circumstances, propulsion does not only arise from poling, but from ski-snow interactions such as gliding and friction. Skiers continuously regulate weight transfer and body orientation to account for these factors. The simulator constrains the skier to a stationary base, thereby reducing the role of balance and lower-limb contributions. Bere et al. showed in 2011 that in alpine skiing, snow, steepness, and velocity might lead to difficulties and raise joint loading as well as injury risk (Bere et al., 2011). Ski boots and equipment also influence joint loading due to their effect on postural control (Noé et al., 2020; Noé et al., 2019).

## Data Availability

The raw data supporting the conclusions of this article will be made available by the authors, without undue reservation.

## Footnotes

1
https://orthoload.com/database/?implantId=12&activityId=228&activityIndentationLevel=3&parameterId=1&parameterIndentationLevel=-1&patientId=hsr&fileId=hsrwt4&fileType=t&selectBox=file
